# The effect of knee resizing illusions on pain and swelling in symptomatic knee osteoarthritis: a case report

**DOI:** 10.1097/PR9.0000000000000795

**Published:** 2019-11-21

**Authors:** Erin MacIntyre, Maja Sigerseth, Brian W. Pulling, Roger Newport, Tasha R. Stanton

**Affiliations:** a Sports and Spinal Physiotherapy, Sunshine Coast, Queensland, Australia; b Department of Global Public Health and Primary Care, Member of Physiotherapy Research Group, University of Bergen, Bergen, Norway; c IIMPACT in Health, The University of South Australia, Adelaide, South Australia, Australia; d School of Sport, Exercise and Health Sciences, Loughborough University, Loughborough, United Kingdom; e Neuroscience Research Australia, Sydney, New South Wales, Australia

**Keywords:** Osteoarthritis, Body illusion, Mediated reality, Mental representation, Pain, Swelling

## Abstract

**Introduction:**

Resizing illusions that manipulate perceived body size are analgesic in some chronic pain conditions. Little is known whether such illusions may also alter other physiological features, such as swelling.

**Objectives:**

To determine the effects of a knee resizing illusion on knee pain and swelling in symptomatic osteoarthritis.

**Methods:**

This case study was extracted from a larger study evaluating the analgesic effects of resizing illusions in people with knee osteoarthritis. A mediated reality system (alters real-time video) was used to provide resizing “stretch” and “shrink” illusions of the knee. Knee pain intensity (0–100 numerical rating scale) was measured before and after illusion and after sustained (3 minutes) and repeated (n = 10) illusions. In this case study, knee swelling (leg circumference below, at, and above the knee) was also measured.

**Results:**

The 55-year-old male participant reported a long history of episodic knee pain and swelling that was subsequently diagnosed as severe osteoarthritis in 2013. In the first testing session, the participant experienced an increase in pain with the shrink illusion and a decrease in pain with stretch illusion. A noticeable increase in knee swelling was also observed. Thus, in sessions 2/3, swelling was also assessed. The stretch illusion decreased pain to the largest extent, but resulted in increased knee swelling. Repeated and sustained stretch illusions had cumulative analgesic effects but resulted in cumulative increases in swelling. While the shrink illusion increased pain, sustained (∼10 minutes) visual minification of the entire knee and leg reduced both pain and swelling.

**Conclusion:**

Our case report suggests that both pain and swelling may be modifiable by altering body-relevant sensory input in symptomatic knee osteoarthritis.

## 1. Introduction

There is growing evidence that body resizing illusions are analgesic in some pain conditions,^4^ including knee osteoarthritis.^21^ Recent theoretical models, namely the cortical body matrix model,^12^ postulate that both pain and physiological regulation (eg, swelling) of the body are intertwined with body perception. That is, a multisensory representation of the body is involved in maintaining integrity and homeostatic regulation of the body, and this body matrix dynamically adapts to changes in (perceived) body structure.^12^ Indeed, visual illusions that distort the size of the affected limb in people with complex regional pain syndrome (CRPS) modulate both movement-evoked pain and swelling.^14^

The effect of body illusions on physiological regulation is particularly relevant in knee osteoarthritis, where fluctuating or persistent swelling is common.^3^ However, CRPS is an atypical pain condition (eg, swelling of the affected limb can be induced by merely imagining movement^11^), making it uncertain whether illusion-induced swelling changes would also occur in osteoarthritis. Therefore, in this case report, we examine the effect of illusory resizing of the painful knee (using mediated reality) on pain intensity and swelling in a patient with symptomatic knee osteoarthritis.

## 2. Methods

This case was taken from our previous knee osteoarthritis illusion study.^21^ The initial protocol and case study were approved by the UniSA Human Research Ethics Board (ID:28496); the participant provided written informed consent for both.

**Figure 1.**
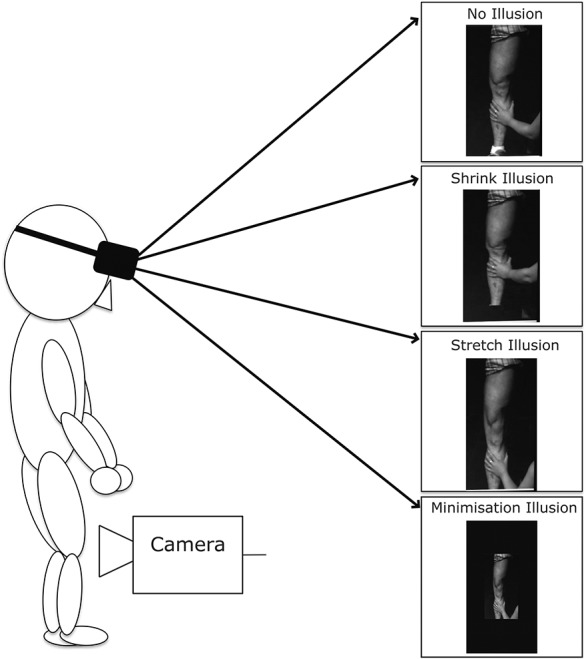
The MIRAGE mediated reality set-up and and experimental conditions.

The participant attended 3 sessions, with resizing illusions performed on his symptomatic knee using the MIRAGE illusion system (tested in standing; Fig. 1). The participant viewed video of his own knee from the front (as if looking in a mirror) through a head mounted display. To create the resizing illusions, video images of the knee were altered using customised LabVIEW software. The knee was visually stretched or shrunk (∼50% of normal size), accompanied by the experimenter applying congruent tactile input on the calf (gentle longitudinal traction or compression, respectively).

Session 1 evaluated whether body illusions produced more analgesia than control conditions. The participant underwent 2 illusion conditions (stretch/shrink: congruent vision-touch) and 6 stretch/shrink control conditions (touch-only, vision-only, incongruent vision-touch), in a randomised order with pain intensity (0–100 numerical rating scale^9^) assessed before and after condition. Each illusion/condition lasted ∼4 to 5 seconds, with the knee returned to normal size for the 2-minute break between each condition.

The illusion that produced the greatest pain reduction was used in sessions 2 and 3. To determine cumulative analgesic benefits, this illusion was sustained for 3 minutes (participant viewed his visually altered knee, rating pain intensity every 30 seconds) in session 2. The illusion was also repeated 10 times (pain intensity rated before/after illusion; 30 seconds break between illusions) in both sessions.

During sessions 2/3, additional procedures were undertaken in this participant. Knee swelling was assessed (before/after illusion), through tape measure, at 3 locations (skin marked for consistency): 1 cm below patella inferior pole; at patella midpoint; and 1 cm above patella superior border. A unisensory illusion of the entire limb (50% visual minification) was also tested (last in each session) and was sustained for 10 minutes (pain/swelling measured before/after illusion).

One-sample *t* tests evaluated if baseline pain scores differed significantly from the postillusion pain scores (for all repeated/sustained illusions).

## 3. Results

A 55-year-old Caucasian man (P.C.) reported a 30-year history of episodic knee pain after a horse-riding accident, where the horse fell and rolled over his left leg. He reported having a sore knee and back, but that the pain subsided over the following weeks. No medical attention was sought. He reported that his knee was without incident until his forties when he began experiencing periods of knee pain and swelling, which was diagnosed as a torn meniscus. He underwent 3 knee arthroscopies for the meniscal injury and reported good outcome (periods without pain/swelling after each procedure). P.C. reported that his knee was asymptomatic until 2013 (∼53 years old), at which time his knee pain and swelling returned after an increase in activity level. His symptoms were managed pharmaceutically with prednisone and methotrexate to good effect, although medication side effects prevented long-term use. He also reported undertaking physiotherapy (exercise/education), acupuncture, and osteopathy. In 2015, he underwent repeat knee radiographs which showed severe osteoarthritis. See Table 1 for participant demographics and session-relevant details.

**Table 1:**
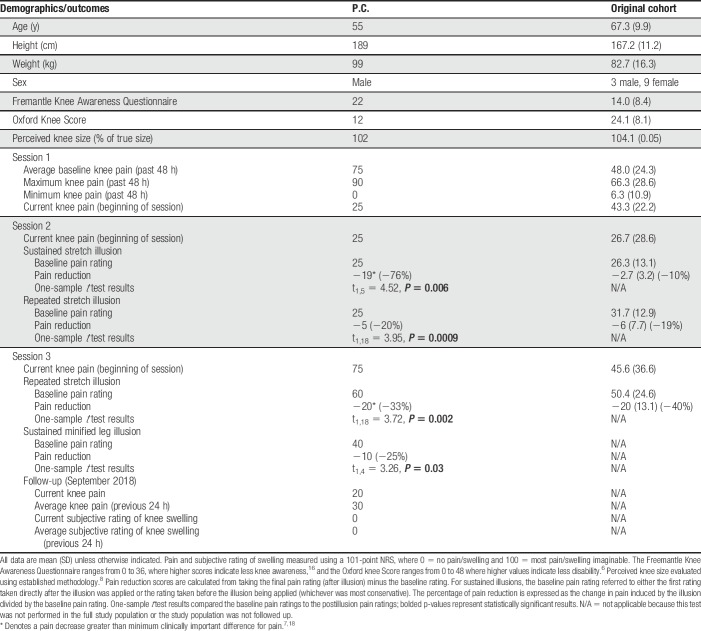
Demographics and pain measures for the case study participant (P.C.) and the original cohort.

In session 1, P.C. had an increase in knee pain (+10) with the shrink illusion and a pain decrease (−5) with the stretch illusion (no change in pain with control conditions). P.C. had a visually noticeable increase in knee swelling post-stretch illusion.

**Figure 2.**
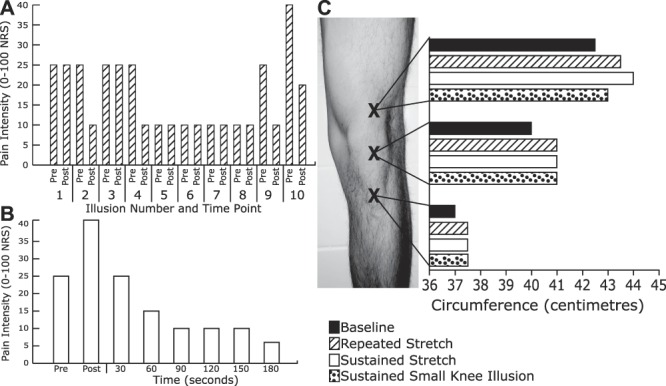
Session 2 results. (A) Pain intensity ratings during repeated stretch illusions; (B) pain intensity ratings during sustained stretch illusion; (C) knee swelling measurements (leg circumference in centimeters) at baseline, after repeated stretch illusions, after sustained stretch illusion, and after a sustained minimised knee/leg illusion. NRS, Numerical Rating Scale.

**Figure 3.**
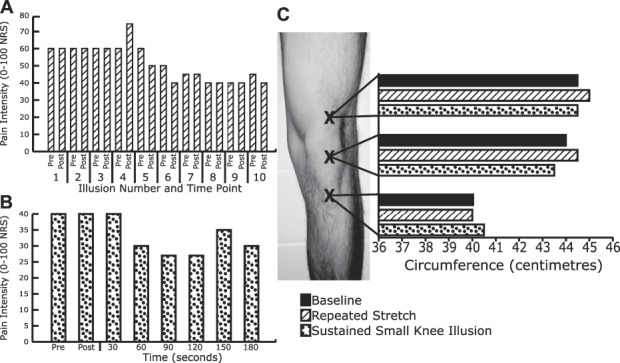
Session 3 results. (A) Pain intensity ratings during repeated stretch illusions; (B) pain intensity ratings during sustained minimised knee/leg illusion; (C) knee swelling measurements (leg circumference in centimeters) at baseline, after repeated stretch illusions, and after a sustained minimised knee/leg illusion. NRS, Numerical Rating Scale.

In sessions 2/3, repeated and sustained application of the stretch illusion both had cumulative effects resulting in increased analgesia (Figs. 2A/B and 3A), but also increased swelling, primarily superior to the patella (Figs. 2C and 3C). P.C. reported transient, but consistent, feelings of nausea during the stretch illusion. In contrast, when P.C. underwent the sustained unisensory minification illusion (reduced overall size of the knee/leg), knee swelling reduced (Figs. 2C and 3C), and pain decreased (measured only in session 3; Fig. 3B). Statistically significant pain reductions were present for all repeated/sustained illusions (Table 1).

Upon follow-up (September 2018, ∼2 years after study), P.C. reported that he underwent a total knee replacement (October 2015), followed by extensive physiotherapy which improved, but did not entirely relieve, his knee pain and swelling (Table 1).

## 4. Discussion

This case study presents the first evidence that body resizing illusions may modulate both pain and swelling in knee osteoarthritis. That the effects on pain and swelling were conflicting during the knee stretch illusion (congruent vision-touch; visually altering the knee), but consistent during a unisensory minimisation illusion (vision-only; visually altering the entire limb), suggests differential pathways of effect based on the type of sensory manipulation.

Past work in CRPS has shown that the perceived anthropometric characteristics of the body (size of the painful limb) are linked to pain^10,14^ and to its physiological regulation, namely swelling.^14^ Such findings, combined with evidence of bidirectional links between body ownership and physiological regulation,^2,13^ have led to the proposal of the cortical body matrix theory.^12^ We extend this theory by showing that altered sensory input can have differential influences on pain and swelling, potentially suggesting unique drivers of each.

Why might the stretch illusion reduce pain but increase swelling (with effects heightened for repeated application) whereas a minimised view of the entire limb reduces both pain and swelling? First, that a sustained stretch illusion (visual change not repeated) also reduced pain and increased swelling (Fig. 2B/2C) suggests against such effects being solely driven by neural processing initiated with viewing the real-time change in body size (eg, via increased visual attention^1^). Second, differences in the type of illusion may be relevant. Illusions differed on the presence/absence of a tactile component, but tactile-only control conditions did not influence pain/swelling suggesting against a simple effect of touch. In addition, the stretch illusion is a nonaffine transformation (site-specific manipulation—the knee itself “stretches”), while a minimised limb illusion is an affine transformation (rigid body alteration of the entire limb). Past work shows alterations in physiological regulation as a function of body ownership^13^ and that loss of body ownership can be analgesic,^17,19,20^ raising the possibility that either illusion may have induced a loss of knee ownership. Nausea was reported only during the stretch illusion suggesting that the knee was not disowned for that condition. Rather, nausea typically occurs when what you see does not match what you feel (sensory-mismatch), with cybersickness heightened as a function of immersion.^23^ The stretch illusion alters the knee itself, inducing an incongruence between the seen and the felt knee which may be sufficient to elicit protective responses such as nausea and increased swelling or, in past work, feelings of disgust (which have not been reported for full limb minimisation).^15^ However, such incongruence is typically algesic^5^ or has no effect on pain,^22^ neither of which was seen here. Instead, perceived body size may also play a role in analgesia. People with knee osteoarthritis have an altered perception of their painful knee^16^—P.C. perceived his knee to be larger than it actually was (2% larger). Research in hand osteoarthritis has shown that regardless of the illusion (stretch or shrink), illusory resizing normalises mental hand representation.^8^ Such findings may support a role of mental representation in the analgesic effects during both enlarging and reducing illusions seen here.

### 4.1. Study limitations

Limb ownership was not evaluated during illusions (purposefully to ensure participant blinding). Future work is warranted to explore the effects of these illusions on ownership and mental representation of the body and to explore phenomenological accounts (does a stretched knee appear swollen? ie, perceived swelling influences actual swelling). In addition, knee swelling measurement over time (without any illusion) was not evaluated, making it possible that swelling would have reduced without a minimised knee illusion. However, that there was no visual change in knee swelling for ∼15 minutes after illusion in session 1 supports the present findings.

## 5. Conclusions

We present the first detailed case study in which body resizing illusions modulate osteoarthritic pain and swelling. Our findings add to the growing evidence that body-relevant sensory input can have profound influences on pain and physiological regulation of the body.

## Disclosures

T.R. Stanton received travel and accommodation support from Eli Lilly Ltd for speaking engagements (2014; unrelated to the present topic). R. Newport is the creator the MIRAGE mediated-reality systems. The University of Nottingham (United Kingdom) received equipment fees for creation of mediated-reality systems for external laboratories. The remaining authors have no conflicts of interest to declare.
